# Mycophenolate sodium treatment in patients with primary Sjögren syndrome: a pilot trial

**DOI:** 10.1186/ar2322

**Published:** 2007-11-06

**Authors:** Peter Willeke, Bernhard Schlüter, Heidemarie Becker, Heiko Schotte, Wolfram Domschke, Markus Gaubitz

**Affiliations:** 1 Department of Medicine B, Muenster University Hospital, Albert Schweitzer Street 33, D-48129 Muenster, Germany; 2 Institute of Clinical Chemistry and Laboratory Medicine, Muenster University Hospital, Albert Schweitzer Street 33, D-48129 Muenster, Germany

## Abstract

The aim of this study was to evaluate the efficacy and safety of mycophenolate sodium (MPS) in patients with primary Sjögren syndrome (pSS) refractory to other immunosuppressive agents. Eleven patients with pSS were treated with MPS up to 1,440 mg daily for an observation period of 6 months in this single-center, open-label pilot trial. At baseline, after 3 months, and after 6 months, we examined the clinical status, including glandular function tests, as well as different laboratory parameters associated with pSS. In addition, subjective parameters were determined on the basis of different questionnaires. Treatment with MPS was well tolerated in 8 of 11 patients. Due to vertigo or gastrointestinal discomfort, two patients did not complete the trial. One patient developed pneumonia 2 weeks after treatment and was withdrawn. In the remaining patients, MPS treatment resulted in subjective improvement of ocular dryness on a visual analogue scale and a reduced demand for artificial tear supplementations. However, no significant alterations of objective parameters for dryness of eyes and mouth were observed, although a substantial improvement of glandular functions occurred in two patients with short disease duration. In addition, treatment with MPS resulted in significant reduction of hypergammaglobulinemia and rheumatoid factors as well as an increase of complement levels and white blood cells. MPS promises to be an additional therapeutic option for patients with pSS, at least in those with shorter disease duration. Further investigations about the efficacy and safety of MPS in pSS have to be performed in larger numbers of patients.

## Introduction

Primary Sjögren syndrome (pSS) is an autoimmune disorder characterized by keratoconjunctivitis sicca and xerostomia. In addition, various extraglandular manifestations may develop. Several immunomodulating agents have been attempted in the treatment of pSS without achieving satisfactory results [1]. Currently, there is no approved systemic treatment for pSS.

Mycophenolic acid (MPA) is a selective inhibitor of inosine monophosphate dehydrogenase which leads to inhibition of the *de novo *pathway of nucleotide synthesis. The antiproliferative effect of MPA mainly affects activated T and B lymphocytes because the proliferation of these cells is critically dependent on the *de novo *purine synthesis compared with other eukaryotic cells [2]. Since these lymphocytes have been suggested to play a pivotal role in the inflammation and immunopathogenesis of pSS [3,4]., MPA might be a promising agent in the treatment of pSS.

MPA-containing compounds such as mycophenolate mofetil (MMF) and enteric-coated mycophenolate sodium (MPS) are immunosuppressive drugs approved for the prevention of transplant rejection [5]. MPS 720 mg and MMF 1,000 mg deliver nearly equimolar doses of the active immunosuppressive agent [6].

MMF is an effective treatment in systemic lupus erythematosus (SLE) [7,8]. and other autoimmune diseases [9,10]. MMF has been used as maintenance therapy after treatment with rituximab (anti-CD20 antibody) in a patient with pSS [11]. We reported a case of successful treatment with MMF in pSS with vasculitis [12]. The recent observations and the immunosuppressive effect of MPA in other autoimmune diseases led us to evaluate the efficacy and safety of MPA treatment in patients with pSS refractory to other immunosuppressive agents.

## Materials and methods

### Study design

We performed a prospective, single-center, open-label pilot trial for an observation period of 6 months. Medical treatment was initiated with one tablet of 360 mg MPS per day. The dosage was increased weekly by 360 mg up to a maximum stable dose of 1,440 mg daily. In patients not tolerating the drug well, the dosage was reduced to 720 mg per day. All patients gave written informed consent to participate. The study protocol was approved by the local independent ethics committee.

### Patient selection criteria

Inclusion and exclusion criteria for the trial are presented in Table 1. Eligibility criteria included the diagnosis of pSS based on the American-European Consensus criteria [13] provided that the patients had evidence of active disease. Since there are no validated disease activity criteria for pSS, active disease was defined by elevated erythrocyte sedimentation rate (ESR) (>25 mm/hour), hypergammaglobulinemia (>1,500 mg/dL) and the presence of autoantibodies (that is, anti-SSA and/or SSB antibodies and/or rheumatoid factor [RF]).

**Table 1:** Inclusion and exclusion criteria

Inclusion criteria	Diagnosis of primary Sjögren syndrome based on the American-European Consensus criteria [13]
	Erythrocyte sedimentation rate of greater than 25 mm/hour and hypergammaglobulinemia (>1,500 mg/dL)
	Presence of anti-SSA and/or SSB antibodies and/or rheumatoid factor
	Requirement of artificial teardrops due to symptomatic sicca syndrome
	Inadequate response or intolerance of prior treatment with hydroxychloroquine and/or azathioprine
	Adequate contraception for females of childbearing potential
	
Exclusion criteria	Age below 18 or above 75 years
	Secondary Sjögren syndrome
	History of cancer, severe infections, or other uncontrolled diseases
	Treatment with concomitant disease-modifying antirheumatic drugs within the last 8 weeks before baseline evaluation
	Prednisolone dose of greater than 5 mg/day or changes of prednisolone dose within the last 4 weeks before baseline
	Use of secretagogues (for example, pilocarpine and civemeline) or medications that potentially diminish exocrine gland function (for example, tricyclic antidepressants and anticholinergic drugs)
	Pregnant or lactating women

### Outcome measures

Clinical visits were performed at baseline, week 12, and week 24. After 4 weeks, an additional visit, including clinical examination and laboratory tests, was performed. Patients were asked about possible adverse events (AEs) and about the daily demand for artificial teardrops. Clinical assessment consisted of a general physical examination, the 28-joint count of tender/swollen joints, and a tender point count (maximum of 18).

#### Functional parameters

The lachrymal gland function was assessed by unanesthetized Schirmer's test [14]. A value of less than 5 mm per 5 minutes was taken as abnormal. In addition, we collected the unstimulated whole saliva throughout a 5-minute period by performing the spitting technique [15]. A flow rate of less than 0.5 grams per 5 minutes was considered as glandular hypofunction.

#### Subjective parameters

Patients were instructed to express the severity of ocular dryness, arthralgia, and fatigue on a 100-mm visual analogue scale (VAS) ranging from 0 for no symptoms to 100 for extreme symptoms. Outcome was also determined by the Short Form 36 (SF-36) questionnaire in eight scales (physical functioning, role physical, bodily pain, general health, vitality, social functioning, role emotional, and mental health) ranging from 0 to 100 [16]. A higher value indicates a higher state of well-being. Aggregated physical component summary score and mental component summary score of the SF-36 were calculated. The SF-36 has been validated for patients with pSS [17]. In addition, the Health Assessment Questionnaire was completed by our patients [18]. Values range from 0 to 3. A lower score indicates better health.

#### Laboratory parameters

Routine laboratory parameters (that is, ESR, C-reactive protein, renal and liver function tests, total protein, and full blood count) were determined at each visit. Levels of immunoglobulins (IgG, IgM, and IgA), IgM-RF, and serum concentrations of complement levels (C3 and C4) were measured by nephelometry (BN2; Dade Behring, now Siemens Medical Solutions Diagnostics GmbH, Bad Nauheim, Germany). IgG antibodies to SSA and SSB were analyzed by fluorescence enzyme immunoassay (Phadia GmbH, Freiburg, Germany). Protein electrophoresis was performed on an Olympus Hite 320 (Olympus-Diagnostika GmbH, Rees, Germany).

### Statistical analysis

Data were analyzed by means of the statistic software package SPSS 12.0 (SPSS Inc., Chicago, IL, USA). The significance of changes from baseline was measured by Wilcoxon test. A *p *value of less than 0.05 was considered significant.

## Results

### Clinical characteristics of patients

Eleven patients with active pSS were included in this study (Table 2). All patients were women with a mean age of 50.1 ± 10.8 years and a mean disease duration of 9.5 ± 5.4 years. All patients had an abnormal Schirmer's test. In six patients, a diminished salivary flow rate was detected. Seven patients had mild leukopenia (<4,000/μL) at baseline evaluation (Table 2). As for other extraglandular manifestations, eight patients had arthralgia, five had Raynaud syndrome, one patient had polyneuropathy, and one had vasculitis.

**Table 2:** Clinical characteristics of patients

Patient	Age in years	Gender	Disease duration in years	Anti-SSA/Anti-SSB/RF	Extraglandular manifestations	Prednisolone (mg/day)	Adverse events
1^a^	59	Female	13	+/+/+	Arthralgia	-	Vertigo, perspiration
2	46	Female	12	+/+/+	LP, RS	-	GI discomfort, common cold
3^a^	50	Female	15	+/+/+	Arthralgia, RS, LP	-	GI discomfort
4	67	Female	17	+/+/+	Arthralgia, RS	2.5	None
5	60	Female	4	+/+/+	Arthralgia, LP, VA	-	GI discomfort^b^
6^a^	61	Female	5	+/+/+	Arthralgia, LP	-	Pneumonia
7	53	Female	13	+/-/+	LP, PNP	2.5	GI discomfort, herpes labialis
8	40	Female	2	+/-/-	Arthralgia, LP	-	None
9	35	Female	12	+/-/+	LP, RS	5	None
10	44	Female	9	+/+/+	Arthralgia, RS	-	Palpitation, perspiration^b^
11	36	Female	2	+/+/-	Arthralgia	-	Common cold

^a^Patients 1, 3, and 6 did not complete the study; ^b^dose was reduced to 720 mg/day after week 12. GI, gastrointestinal; LP, leukopenia; PNP, polyneuropathy; RF, rheumatoid factor; RS, Raynaud syndrome; VA, vasculitis.

### Safety of mycophenolate sodium

Eight patients completed the study period of 24 weeks. Two patients were unwilling to continue the study after week 4 due to vertigo (patient 1) and gastrointestinal (GI) complaints (patient 3). Patient 6 was withdrawn at day 15 after developing pneumonia that caused hospitalization. The patient fully recovered after antibiotic treatment. This event was the only serious AE. The reported AEs possibly related to the study medication were of mild intensity. GI discomfort (defined by the occurrence of nausea, dyspepsia, or diarrhea) was the most frequent AE in 4 of the 11 patients included (Table 2). These patients had no previous evidence of intestinal involvement associated with the disease. In one patient, a herpes labialis infection occurred and was controlled by local application of aciclovir. Two patients during the study developed a common cold that required no additional medical treatment. MPS was reduced in patients 5 and 10 after week 12 due to mild GI discomfort and palpitation, respectively, which resolved after a dose reduction of MPS. We observed no significant changes in body weight, blood pressure, or heart rate. Also, no drug-related hematological abnormalities were observed.

### Changes in outcome parameters

Outcome parameters were evaluated for the eight patients who completed the study (Table 3). No significant changes in the Schirmer's test or the amount of unstimulated whole saliva in the cohort were observed. However, in patients 8 and 11, who had a relatively short disease duration, a significant improvement was observed in both the Schirmer's test (1.25 ± 0.35 mm at baseline versus 6.75 ± 5.3 mm after 24 weeks) and the unstimulated whole saliva (0.17 ± 0.22 grams per 5 minutes at baseline versus 0.44 ± 0.11 grams per 5 minutes at week 24).

**Table 3:** Baseline values and changes in efficacy parameters

Parameter	Baseline	Week 12	Week 24
Glandular function tests			
Schirmer's test (millimeters per 5 minutes)	2.0 ± 3.2	3.4 ± 3.5	4.4 ± 4.9
Whole saliva (grams per 5 minutes)	0.49 ± 0.41	0.48 ± 0.41	0.56 ± 0.41
Laboratory tests			
Erythrocyte sedimentation rate (mm/hour)	47.6 ± 20.5	44.1 ± 23.3	44.2 ± 21.0
Gamma globulins (g/L)	21.3 ± 6.3	19.5 ± 8.6^a^	18.8 ± 7.5^b^
IgG (mg/dL)	2,159 ± 949	2,048 ± 755	2,025 ± 895
IgM (mg/dL)	189 ± 108	144 ± 62.7^a^	142 ± 62.8^a^
IgA (mg/dL)	399 ± 417	359 ± 363	329 ± 305
Rheumatoid factor IgM (IU/mL)	275 ± 504	193 ± 380	179 ± 317^a^
Anti-SSA antibodies (U/mL)	5,863 ± 5,321	6,308 ± 4,008	5,892 ± 8,859
Anti-SSB antibodies (U/mL)	1,217 ± 1,386	1,424 ± 1,925	1,320 ± 1,668
C3 (mg/dL)	99.3 ± 30.2	101 ± 33.5	108 ± 34.7^b^
C4 (mg/dL)	14.9 ± 8.8	16.4 ± 9.5^a^	16.8 ± 8.9^b^
Subjective findings			
VAS sicca syndrome (0 to 100 mm)	68.7 ± 15.6	56.4 ± 19.9	52.8 ± 20.8^a^
VAS arthralgia (0 to 100 mm)	62.1 ± 23.4	45.1 ± 34.3	47.5 ± 30.0
VAS fatigue (0 to 100 mm)	63.4 ± 24.0	57.4 ± 33.4	65.3 ± 22.6
Use of artificial teardrops (times per day)	3.7 ± 2.3	1.8 ± 1.3^a^	1.6 ± 1.9^b^
Health Assessment Questionnaire score	0.73 ± 0.77	0.81 ± 0.84	0.73 ± 0.80

^a^*p *< 0.05 versus baseline value analyzed by Wilcoxon signed rank test; ^b^*p *< 0.02 versus baseline value analyzed by Wilcoxon signed rank test. Table presents outcome parameters in the eight patients with primary Sjögren syndrome who completed the study. Data are presented as mean ± standard deviation. Ig, immunoglobulin; VAS, visual analogue scale.

No changes in the 28-swollen/tender joint count or in the number of tender points were observed (data not shown). In one patient with vasculitis, a remarkable improvement of the vasculitis of the lower arms and legs was observed after 12 weeks (patient 5). This improvement lasted throughout the study. No significant changes concerning the Raynaud syndrome were observed, although an angiologic examination was not performed as follow-up procedure.

#### Subjective parameters

Significant improvement in patients' assessment of ocular dryness on a VAS was observed (*p *< 0.05). The demand for daily artificial tear supplementation decreased significantly during treatment (*p *< 0.02). No significant improvement in the other VAS was observed.

**Figure 1:**
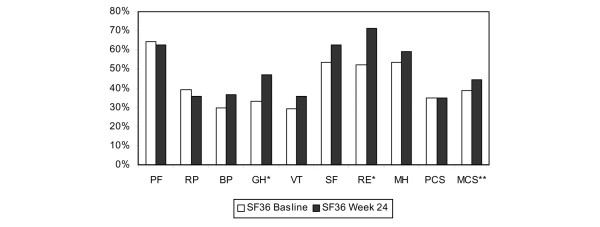
Short Form 36 (SF-36) at baseline and after 24 weeks of treatment with mycophenolate sodium in patients with primary Sjögren syndrome (*n *= 8). The SF-36 domains are physical functioning (PF), role physical (RP), bodily pain (BP), general health (GH), vitality (VT), social functioning (SF), role emotional (RE), mental health (MH), and the physical and mental component summary scores (PCS and MCS). The GH and RE domains increased significantly (*p *< 0.05) after 24 weeks (*). The increase of the MCS did not reach significance (**p = 0.06).

Patients had significant improvement in the general health and the role emotional domains of the SF-36 (Figure 1). Other domains and the physical component summary score did not improve significantly. Although a clear tendency toward improvement of the mental component summary score was observed, statistical significance was not reached (*p *= 0.06).

#### Laboratory parameters

We detected a significant reduction of gamma globulins after 12 and 24 weeks of treatment with MPS (*p *< 0.05). Also, a significant reduction of IgM was observed after 12 and 24 weeks, whereas the reduction of IgG or IgA was not significant. No significant change in ESR was measured. A significant increase of both C3 and C4 complement levels occurred during the treatment. We further detected a decrease of IgM-RF after 24 weeks. The white blood cell count increased significantly from 4,478 ± 1,190 cells per microliter at baseline to 5,703 ± 1,508 cells per microliter after 24 weeks (*p *< 0.05) (data not shown). No differences were found in red blood count, thrombocytes, or renal or liver function parameters.

## Discussion

We present the first controlled pilot trial of MPS treatment in patients with pSS. Our study shows that MPS can improve symptoms and laboratory findings in patients with active pSS. The optimum systemic treatment of pSS is still unclear. Although no controlled study has been performed so far, MPA was suggested as sole or adjuvant treatment for pSS in a recent review [19].

We found a significant reduction of ocular dryness assessed by a VAS. In accordance with this finding, the daily demand for artificial teardrops decreased significantly in our patients. On the other hand, no improvement in the salivary and lachrymal gland functions in our cohort was observed. It should be noted, however, that we observed a remarkable improvement of glandular function in two patients with a disease duration of less than 3 years, possibly due to recovery of the glandular tissue. It has been reported that regeneration of glandular tissue and recovery of glandular function can occur only in patients with residual glandular function [20]. Lack of improvement in the other patients might be due to irreversible damage of the glandular tissue after a long disease duration. Improvement of salivary gland function has been reported in patients with early pSS after treatment with rituximab, an anti-CD20 monoclonal antibody. These observations emphasize the need for an earlier and more aggressive treatment in pSS patients with short disease duration [21].

Mycophenolate has been used in systemic vasculitis [22]. In the one patient with vasculitis, a reduction that has been reported previously in a pSS patient treated with MMF was observed [12]. Thus, MPA-containing compounds might be useful in this systemic manifestation.

We found a significant reduction of gamma globulins during treatment. Hypergammaglobulinemia has been proposed as a suitable target for therapy and as a primary outcome measure for the evaluation of the pSS treatment [23]. It has been suggested that reduction of B-cell hyperactivity with immunosuppressants might be the best prevention of lymphoproliferation in pSS [24]. As a low level of C4 has been associated with an increased risk of developing lymphoproliferative disease [25] and as our data show an increase of C3 and C4 levels in patients treated with MPS, this drug may be essentially beneficial in this context.

Furthermore, we found a significant reduction of IgM after 24 weeks, whereas no substantial reduction of IgG or IgA was detectable. In addition, a reduction of IgM-RF during the treatment was observed. Treatment with rituximab has also been accompanied by a decrease of IgM and IgM-RF in pSS patients without changes in IgG or IgA levels [21,26].

We found no changes in anti-SSA/anti-SSB antibody titers. These antibodies are relatively stable over time in individual patients and thus are not suggested as an outcome measure of disease activity [27]. Likewise, in pSS patients receiving B-cell-depleting agents (for example, rituximab or epratuzumab), no changes in anti-SSA and anti-SSB antibodies were detected [26,28].

We observed an increase of leukocytes/neutrophils during MPS treatment. Leukopenia is one of the most frequent extraglandular manifestations of the disease and is probably mediated by antineutrophil antibodies [29]. Leukopenia in pSS has been shown to be reversed by immunosuppressive treatment with corticosteroids or hydroxychloroquine [29]. Our data show that MPS also might be effective in treating pSS-associated leukopenia.

Patients of our cohort showed significant improvement in the general health and role emotional domains of the SF-36. A trend toward a significant increase of the mental component summary score was observed. This indicates an improvement in psychological distress, social disability due to emotional problems, and self-related health [16].

The overall tolerability of MPS in patients with pSS was acceptable. The most frequent AE was mild GI discomfort. GI discomfort has been reported as the most common AE leading to discontinuation of therapy in transplant recipients [6]. In SLE patients treated with MMF, GI-related symptoms are common as well. The symptoms tend to be mild and can improve with dose reduction [8]. In transplant recipients treated with enteric-coated MPS, less severe GI AEs have been observed as compared with MMF [30].

One of our patients, after 15 days of treatment, developed pneumonia that caused hospitalization. Although the patient received only a fairly low dose of 720 mg MPS per day for only 2 weeks, the event was possibly related to the study drug. All in all, however, compared with studies with transplant recipients [6], the incidence rate of infections was low in the present study.

## Conclusion

Our findings of this open-label pilot trial in patients with pSS suggest that MPS might improve subjective glandular and extraglandular manifestations as well as some laboratory parameters. MPS promises to be an additional therapeutic option in patients with pSS, particularly in those with early disease. Controlled studies including larger numbers of patients with shorter disease durations are necessary to assess more comprehensively the efficacy and safety of MPS in pSS.

## Abbreviations

AE = adverse event; ESR = erythrocyte sedimentation rate; GI = gastrointestinal; Ig = immunoglobulin; MMF = mycophenolate mofetil; MPA = mycophenolic acid; MPS = mycophenolate sodium; pSS = primary Sjögren syndrome; RF = rheumatoid factor; SF-36 = Short Form 36; SLE = systemic lupus erythematosus; VAS = visual analogue scale.

## Competing interests

The authors declare that they have no competing interests.

## Authors' contributions

PW participated in the data analysis and in the design of the study and drafted the manuscript. MG, HS, and HB helped with data collection, patient recruitment, and the design of the study and helped to edit the manuscript. WD helped to edit the manuscript. BS participated in the design and helped in the statistical analysis. All authors read and approved the final manuscript.
